# Shifting the narrative: equity, evidence, effectiveness, and innovation in the era of Ending the HIV Epidemic

**DOI:** 10.1186/s12939-022-01801-6

**Published:** 2022-12-21

**Authors:** Alexander Perez, Rosy Galván, Milanes Morejon

**Affiliations:** National Alliance of State and Territorial AIDS Directors (NASTAD), 444 North Capitol St NW, Suite 339, Washington, DC USA

**Keywords:** Equity, Social justice, Evidence-based, Research

## Abstract

**Background:**

The use of evidence-based (EB) and evidence-informed (EI) criteria in determining the effectiveness of health interventions has been widely adopted by national and international agencies in their attempt to address health gaps, particularly around Ending the HIV Epidemic (EHE) initiatives. Utilization of these rigorous standards has proven critical in making progress towards achieving EHE goals, yet many communities remain unreached and underserved despite widespread adoption of EB/EI standards in public health research and practice. Although a crucial tool for innovative healthcare delivery, emphasis on the use of EB/EI parameters has created bias within the cycle of knowledge creation that favors well-resourced institutions given their capacity to meet the rigorous evaluation standards required of EB/EI science. This bias can systematically exclude institutions more aligned with community needs, such as community-based organizations and other grass-roots initiatives, which may have long-standing interventions that more effectively engage marginalized groups but do not have the capacity to meet EB/EI standards.

**Main body:**

This paper will explore the manifestation of systematic bias and research inequity in the process of identifying and assessing EB/EI HIV care interventions through the lens of a Health Resources and Services Administration funded initiative, coined the Center for Innovation and Engagement, which supports people living with HIV in the United States. An overview of the initiative is provided along with examples of how promising interventions with positive outcomes for members of marginalized communities are excluded in place of interventions that meet traditional standards of scientific rigor but are not novel or particularly innovative. Themes around academic imperialism and power hierarchies will be considered along with key barriers, lessons learned, and recommendations for promoting more equitable EB/EI research practice.

**Conclusions:**

It is crucial for entities supporting public health interventions to prioritize equity and inclusion in all stages of funding, design, and implementation. This is particularly true for conditions, such as HIV, that disproportionally impact the most marginalized. This will require approaching EB/EI research with a critical lens towards power and a willingness to dismantle historical dynamics that perpetuate inequities as a way of encouraging truly innovative solutions to support those who need it most.

## Introduction

The use of evidence-based (EB) and evidence-informed (EI) criteria in gauging the effectiveness of human immunodeficiency virus (HIV)/acquired immunodeficiency syndrome (AIDS) prevention and care interventions has provided crucial insight into the application of findings for real-world settings, particularly in the realm of implementation science, which examines ways to deliver innovative health tools to people living with HIV (PLWH) and populations disproportionately impacted by the HIV epidemic [1, 2]. This has resulted in wide adoption of EB/EI approaches by diverse agencies to address health equity gaps required to meet Ending the HIV Epidemic (EHE) initiative goals in the United States (U.S). While EB/EI approaches have undeniably helped advance health equity, many communities remain underserved and pervasive gaps in engagement and retention in HIV care continue to widen [3]. Although many of these disparities are known to be rooted in structural, social, and behavioral barriers unique to the intersecting identities of each community, many heavily circulated EB/EI interventions are not well-tailored to address these needs or remain inaccessible to organizations with long-standing relationships to these communities [4–10].

The use of EB/EI criteria in HIV prevention and care represents the long-standing use of critically rigorous standards within scientific research as a way of not only measuring the impact of a given approach but, more broadly, ensuring minimal bias is introduced throughout the process of evidence determination [11, 12]. However, there is limited data on the ways in which the evidence determination criteria used to identify and measure EB/EI approaches may disproportionately support interventions implemented by universities and other well-resourced institutions. This bias can generate inequities by prioritizing knowledge created by organizations with the capacity and resources needed to develop, evaluate, and disseminate EB/EI interventions [9]. This may result in the systematic exclusion of innovative models developed by organizations with fewer resources that are more aligned with community needs, such as community-based organizations (CBOs) and other community-led initiatives. These organizations often have long-standing histories of working in historically marginalized communities impacted by HIV but may not have the necessary capacity to evaluate their services with an EB/EI lens or conduct academically rigorous research. Even with well-established recognition of the importance of partnering with CBOs and grassroots organizations to develop culturally responsive approaches to care, funding for programs to identify and disseminate EB/EI approaches may continue to propagate inequities by leaving power imbalances unchallenged rather than prioritizing ways to build the capacity of CBO's and community-led health centers to evaluate existing practices and lead innovative efforts [13–15]. To accelerate the end of the HIV/AIDS epidemic, it is integral to acknowledge and address the disproportionate access to resources for evaluating approaches supporting optimal health for people who have been impacted by the epidemic.

This article will explore how systematic biases and research inequities manifest in the process of identifying and assessing EB/EI HIV care interventions through the lens of a Health Resources and Services Administration (HRSA)-funded Special Projects of National Significance (SPNS) initiative: Evidence-Informed Approaches to Improving Health Outcomes for People Living with HIV. Themes showcasing elements of academic imperialism will be highlighted along with key barriers to uplifting promising practices, lessons learned from the process, and recommendations to promote equitable EB and EI research practice. The SPNS initiative, coined the Center for Innovation and Engagement (CIE), is led by NASTAD in collaboration with Northwestern University’s Center for Prevention Implementation Methodology and Howard Brown Health Center. NASTAD is a leading non-partisan non-profit association that represents public health officials who administer HIV and hepatitis programs in the U.S. NASTAD’s mission is to advance the health and dignity of people living with and impacted by HIV/AIDS, viral hepatitis, and intersecting epidemics by strengthening governmental public health and community partnerships. CIE goals include identifying, cataloging, and disseminating EB/EI approaches to improve health outcomes for PLWH in the U.S.

## Bias in evidence-based (EB) and evidence-informed (EI) practice

EB and EI parameters have been revered as a cornerstone of medical practice after its conception in the 1980s as clinical medicine became increasingly more reliant on data and existing academic knowledge [12]. These parameters provide practitioners and researchers with a framework to address bias or systemic error introduced into a process that may produce a prejudiced outcome [12]. Defined as “the conscientious, explicit, and judicious use of current best evidence” in clinical decision making, the use of EB and EI parameters is meant to leverage clinical expertise and objective data in clinical decision-making [11]. Given this marriage between subjective expertise and objective best practices, EB/EI approaches currently serve as a gold standard when considering novel strategies for addressing ongoing issues across the HIV continuum of care [16]. Yet, an emphasis on clinical outcomes can prioritize the perspectives of highly trained practitioners or researchers over those more closely aligned with the needs of populations that have been historically marginalized, hindering the innovation needed to tackle the very issues these practices work to address.

Engagement and retention in HIV care is now well recognized as embedded within a broader socio-ecological framework that cannot be addressed with biomedical interventions alone [17]. This has caused a gradual ideological shift in contemporary understanding of what “innovative solutions” can mean for addressing ongoing gaps in the engagement and retention of PLWH in care, particularly in the era of EHE. Intersecting issues such as stigma, systemic racism, unstable housing, confidentiality concerns, health literacy, transportation, education, substance use, and unemployment consistently surface across contemporary literature as persistent barriers to care for the most marginalized PLWH [17–21]. This has prompted a concentration of EB/EI research initiatives intentionally aimed at addressing these barriers, which are frequently led by, or substantially dependent on, universities and academic institutions, given their capacity to conduct the high-level research and analysis expected in EB/EI practice. This is further reinforced by funding entities, such as state and federal governments, who rely heavily on these same universities to find innovative solutions for persisting problems due to their well-established track record of producing evidence-based results. Ultimately, this creates a monopolization of intervention development, manifesting as a form of “academic imperialism,” where well-resourced institutions led by highly-trained experts are granted opportunities to explore issues and develop solutions for populations that are rarely represented within the institutions themselves [22].

“Academic imperialism” can only be defined by first understanding the term “imperialism,” and it’s varied manifestations. Imperialism can be defined as “the practice, theory, and the attitudes of a dominant metropolitan center ruling in a distant territory” and has often been used when discussing political, social, and economic power dynamics between nations [23]. Manifestations of imperialism using this definition have traditionally focused on ongoing western influence in post-colonial nations through dependency on colonial political and economic infrastructure [23]. More recently, there has been an emphasis on the ways in which western thought has dominated knowledge in these same nations, which has been coined as the term academic imperialism [23]. Many of the same power schemas apply to our reliance on universities for the validation and creation of knowledge frameworks in public health. For example, although a wide variety of academic journals exist across the world, the foundation of global academic knowledge is biased towards contributions from “high impact” academic journals, most of which operate in high-income countries (e.g., the United States, European Nations, etc.) and favor research produced from institutions in these same contexts [24]. This bias is an extension of the intellectual authority claimed by colonial powers as the developers of standards for “good” research, which are the standards most journals continue to use today [24]. This has resulted in a staggering inequality in academic thought that favors the ideological constructs of well-resourced nations, which have the means to meet the rigorous standards they themselves developed, over those with fewer resources, which also tend to be nations composed predominantly of communities of color [24]. The same bias continues to be propagated within the domestic public health response, particularly in EB/EI research, which still uses an imperialistic framework when seeking to develop new ways to solve ongoing problems, such as the HIV/AIDS epidemic.

Although interventions created through traditional academic avenues continuously prove to be critical tools in HIV public health practice, the imperialism propagated through the unchecked power dynamics inherent to the prioritization of EB/EI approaches has routinized the recycling of well-established interventions for engagement and retention in care under the guise of innovation. This was made evident through NASTAD’s CIE project, in which results from a systematic literature review of interventions were prioritized over data collected at several HIV-related conferences, and a broadly cast request for information (RFI) sent to health departments, CBOs, and AIDS Service Organizations (ASOs) across the U.S. The intervention identification process is summarized in the sections that follow.

## Methods: intervention identification process

### Literature review

A systematic literature review was conducted by Northwestern University to identify evidence-based and evidence-informed approaches and interventions that are designed to engage PLWH who are not receiving or who are at risk of not continuing to receive HIV healthcare. The literature review consisted of several phases of targeted review and exclusion to produce a set of manuscripts describing interventions with *linkage*, *re-engagement*, and *retention* in care outcomes. For this review, *linkage* was defined as the initiation of HIV-related care (e.g., medical, psychological, social service-related) for people newly diagnosed with HIV or those that were previously diagnosed and never began treatment; *re-engagement* as a continuation of treatment or services for those diagnosed with HIV that have fallen out of care; and *retention* as regular engagement in care after being linked to or re-engaged in care. Included manuscripts should describe studies focused on the effectiveness of a clearly defined intervention or approach and describe linkage, retention, and/or re-engagement outcomes, not including HIV testing interventions with a passive referral or an inadequately described linkage component.

**Fig. 1:**
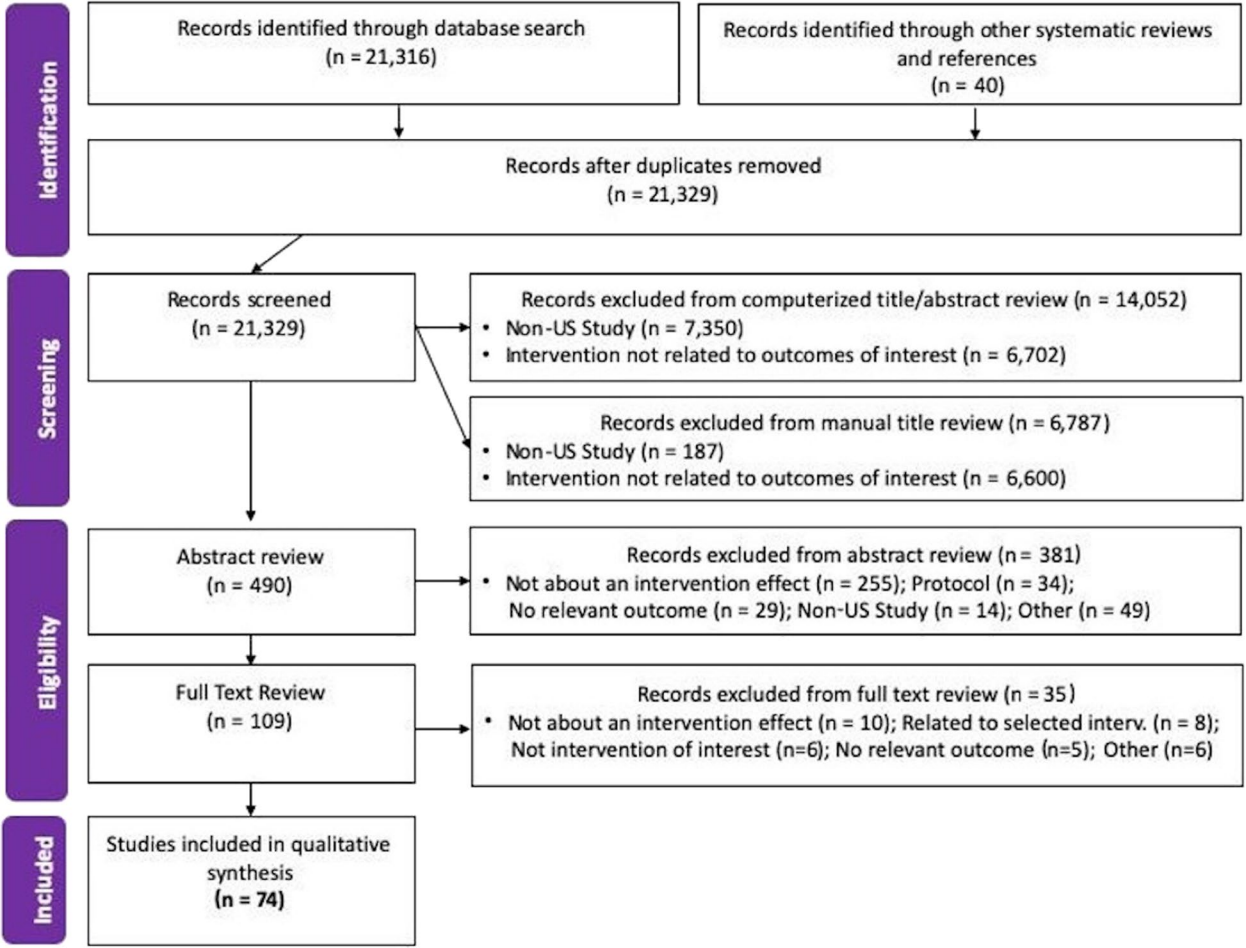
PRISMA Chart Describing Literature Review Intervention Identification Process for Consideration into CIE Compendium

A research librarian developed a comprehensive search strategy in collaboration with the review authors. Searches were conducted on December 7, 2018, in the following databases: PubMed MEDLINE, Embase (Elsevier), Cochrane Library and Cochrane Register of Controlled Trials (CENTRAL) (Wiley), PsycINFO (Ebsco), and Web of Science. The PubMed MEDLINE search was adapted for all additional databases using database-specific logic and wildcards. All databases were searched back to 2005, and an English language filter was applied. The search strategy included terms related to 1) people living with HIV/AIDS; 2) treatment uptake, adherence, and retention; and 3) intervention, implementation, and evaluation. As this review was interested in interventions in the United States, citations that discussed countries other than the United States without mention of the United States were excluded using the Boolean NOT feature. This search yielded a total of 21,302 results across all databases after de-duplication. Eligible studies for analysis were identified using a combination of automatic and manual screening processes, as described in Fig. 1.

### Request for information (RFI) survey

**Fig. 2:**
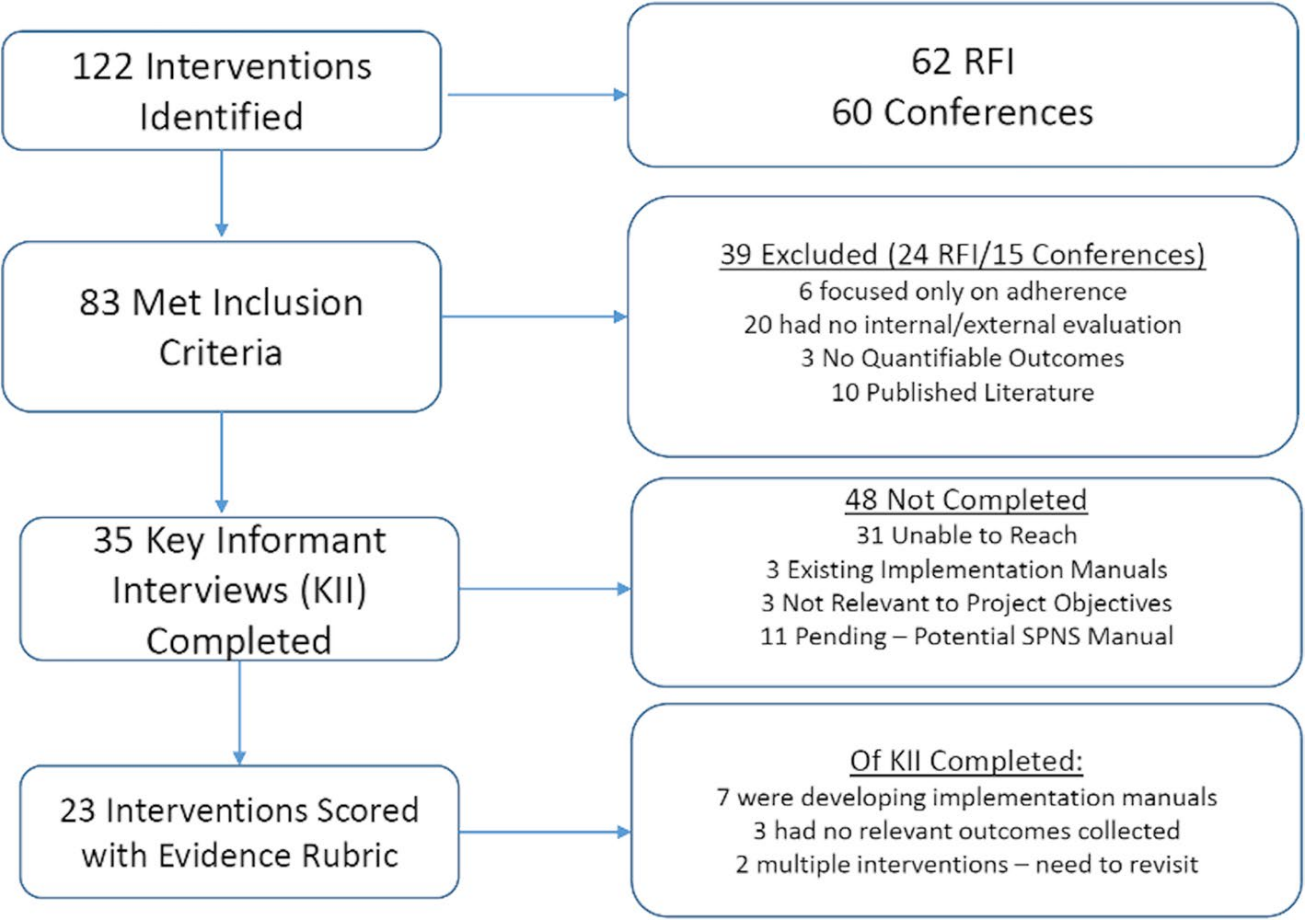
Flow Diagram of Request for Information Process to Identify Interventions for Consideration into CIE Compendium

NASTAD developed an RFI survey to identify interventions that have not been published in academic literature using the same definitions described in the literature review process. The RFI was broadcasted through NASTAD’s membership network in conjunction with tailored outreach to a list of over 150 CBOs and ASOs across the country who work closely with marginalized groups, including lesbian, gay, bisexual, transgender, queer, intersex, and asexual (LGBTQIA+) populations as well as racial and ethnic minorities. Data on interventions were also collected using the same RFI structure via conference presentations (posters and oral presentations) from the 2018 National Ryan White Conference on HIV/AIDS, the 2019 National HIV Prevention Conference, 2019 Conference on Retroviruses and Opportunistic Infections, and 2019 SYNChronicity Conference. NASTAD identified 122 interventions, of which 83 met the inclusion criteria for key informant interviews to collect more information on outcomes, as described in Fig. 2.

### Evidence rubric, impact score, and evidence and dissemination expert panel

Interventions selected from the literature review and the RFI processes were scored using an evidence rubric developed by Northwestern University in collaboration with HRSA and the Centers for Disease Control and Prevention (CDC). The evidence-based criteria established by the Agency for Healthcare Research and Quality and the CDC was used as a foundation for defining EB, and EI approaches. The rubric used a set of criteria to measure the strength and impact of evidence for each intervention to identify approaches, strategies, or models that are proven effective or show promise for improving the care and treatment for PLWH. The criteria used to measure each intervention’s level of evidence included quality of study design (e.g., study design, sample size, time to follow-up), the quality of intervention implementation and analysis (e.g., statistical testing, controlling for confounders, limitations), the strength of the evidence (e.g., positive intervention effects, control arms), and additional limitations and strengths not captured by other rubric components. Items within these categories were scored to determine whether the criteria were met and, where relevant, if the criteria were met with greater qualifications (e.g., larger sample sizes, larger effect size, a greater number of outcomes considered, improvement in clinical outcomes).

In addition to the evidence rubric, an evidence and dissemination expert panel was coordinated to review the final list of interventions that met the evidence threshold. Members were asked to review the evidence score for each intervention along with a summary of its methodology and outcomes. Through a series of discussions, each expert panel member assigned the intervention an “Impact Score” using a series of categories aimed at gauging the real-world impact of the intervention (e.g., feasibility, relevance, acceptability, sustainability, etc.). Final prioritization of interventions for inclusion in the CIE compendium was determined by the weight of the evidence score and the impact score, along with supplementary feedback from expert panel members. Only one intervention identified through the RFI was included in the final list of interventions for the CIE compendium, while the remaining interventions were identified through the literature review led by Northwestern University.

### CIE compendium

The final CIE compendium consisted of 15 interventions: 1 focused on linkage to care, 2 focused on re-engagement, 9 focused on retention, and 3 focused on a combination of linkage, re-engagement, and/or retention. The majority of interventions constituted innovative service delivery models. NASTAD worked with intervention developers to create detailed implementation guides for 10 of the 15 interventions to serve as replication tools for diverse healthcare settings. Fact sheets were developed for the remaining 5 interventions highlighting key considerations from respective academic manuscripts. Implementation guides, intervention summaries, and supplementary replication resources (tip sheets, cost calculator, videos, etc.) were housed on the CIEhealth.org microsite hosted by NASTAD.

## Lessons learned

The process of identifying EB/EI interventions for the CIE compendium highlighted several important considerations for identifying innovative EHE solutions: 1) intervention developers and implementors responding to the RFI had strong anecdotal and clinical evidence suggesting successful uptake of their approaches by patients, yet did not meet the rigorous evaluation standards required for inclusion in the CIE compendium, 2) all interventions selected for the CIE compendium were published in academic journals with the exception of one identified through the RFI, which was in the process of submitting for publication, 3) all interventions were led or substantially supported by universities or academic medical centers, 4) 8 of the 15 final interventions in the compendium already had some form of existing implementation content (e.g., replication guides and/or tools), and 5) the majority of expert panel members were White, cisgender, and highly trained in academic research and/or medical practice. Most importantly, this process uncovered biases embedded in the EB/EI identification process that reinforce academic imperialism and propagate systemic racism in the way evidence and expertise are prioritized.

Examining the implications of using EB/EI evidence-hierarchies in the development of the CIE compendium requires a critical eye for the way evidence is prioritized. A number of interventions identified through the RFI had clinical outcomes that were deemed “promising” by the clinical providers implementing them based on both quantifiable improvements in individual health outcomes and anecdotal feedback from patients. However, none of these implementors were able to rigorously evaluate their work to the statistical degree necessitated by EB/EI criteria for a variety of reasons, including constraints around funding and human resources. For example, ‘Girlfriends Connect,’ an intervention identified through NASTAD’s RFI process, did not make it into the CIE compendium despite reporting 100% linkage when evaluated. The intervention focused on incarcerated transgender women and was the first culturally relevant and theory-based adaptation of the evidence-based intervention, Project START, which is a well-known initiative that met the criteria for inclusion within the CIE compendium despite having already been widely disseminated. The lack of inclusion of an intervention like Girlfriends Connect, even when it was built on the same evidence-based foundation as Project START and provided promising outcomes for a highly marginalized community, highlights how over-reliance on robust statistical metrics can overshadow the value of approaches that may be better suited to address barriers for those most in need. This also exemplifies a dynamic where power is continuously held by institutions, many of which are predominately White academic institutions, that are well-resourced enough to offer the rigor necessary for EB/EI evaluation. Ultimately, this reinforces their ability to secure additional funding and continuing leading the development of EB/EI approaches, despite sometimes recycling well-known interventions under the guise of “innovation”.

Similarly, the concept of “expertise” becomes of particular importance. Although emphasis on clinical expertise and experience is crucial, it is also important to further deconstruct how expertise was defined in the coordination of CIE’s expert panel and how this may have introduced biases into the decision-making process. The evidence rubric developed by Northwestern University considered the ways interventions addressed bias through their design and analytic methods. Yet, no effort was made to address implicit bias amongst expert panel members when prioritizing interventions for the final CIE compendium. Characteristics such as a patient’s race, ethnicity, gender, drug use, socioeconomic status, and HIV/AIDS status have been highlighted as issues that contribute to biases among healthcare professionals in the U.S., and evidence has shown that physicians and nurses manifest implicit bias to a similar degree as the general population [25, 26]. The composition of CIE’s expert panel (e.g., predominantly White, cisgender, women) brings into question how these biases may have manifested in the decision-making process for intervention prioritization given that the sociodemographic characteristics of physicians and nurses (e.g. gender, race, years of experience, location of medical training) are also correlated with levels of bias as exemplified in one study which found significant pro-White bias among internal and emergency medical residents even though no explicit preference for race was reported [25, 26]. Despite the rich experience and expertise offered by CIE’s expert panel, prioritizing the perspectives of mostly White, cisgender, and highly-educated individuals over those of community members who are most impacted by the interventions in question may have influenced the intervention prioritization process. It also highlights a form of systematic racism in which the experiences of those with power and privilege are deemed a more legitimate form of expertise than individuals with actual lived experience.

The marriage between subjective clinical expertise, high-quality external evidence, and subjective community expertise functions as a strong foundation through which public health can explore innovation by expanding its approach to EB/EI science beyond a steadfast dedication to “conventional” research and evaluation frameworks. For example, the CIE evidence rubric may have benefited from weighing certain aspects of a study design or quality (e.g., statistical power) differently if the study focused on a historically marginalized group, reported positive quantitative outcomes, and included quality qualitative data that supported the impact of the intervention even if it did not meet traditional statistical thresholds. This would have prioritized interventions that showed positive community impact through a variety of data points rather than focusing solely on improvements based on a numerical value. Reexamining evidence criteria may require relinquishing traditional structures constituting the creation and use of EB/EI practices, such that they would depend on a wider range of cultural parameters rather than relying solely on the expertise of the clinician and predefined statistical thresholds [27, 28]. Allowing leadership from patients at all levels of the EB/EI process can help to balance some of the inherent biases in decision-making and evaluation, regardless of the level of expertise or familiarity in a given discipline, by introducing a broader perspective through which to identify and mitigate those biases [27]. Similarly, a critical analysis of the established structures that propagate knowledge monopolies and academic imperialism in the creation of EB/EI strategies is required to readily identify where systemic biases are perpetuated. With this in mind, it comes as no surprise that findings from NASTAD’s CIE project would highlight and inevitably reinforce the research inequity inherent to intervention development and evaluation.

## Recommendations for equitable EB/EI research practice

Based on the lessons learned from NASTAD’s CIE project, the below list of recommendations has been compiled to aid future projects in prioritizing equity and inclusion when implementing EB/EI approaches for HIV prevention and care.

i. **Acknowledge and Mitigate Privilege and Power:** Researchers and funding entities engaged in EB/EI model development and implementation should analyze their work within the lens of privilege, White supremacy, and existing power hierarchies. Acknowledge that traditional research avenues utilized to develop, implement, and evaluate interventions for PLWH remain largely inaccessible and often exclude Black, Indigenous and People of Color (BIPOC) who can help reimagine ineffective and inequitable systems. This requires asking key questions to ensure privilege and power are acknowledged and addressed throughout each step of the research process. Some questions to consider include [29]:

1. Who is impacted, positively or negatively, by the issue you plan to study? How are they represented on your research team?
2. What are the causal factors or root causes of the issue, and how are research questions informed by these root causes?
3. How are community values represented in the research question(s)?
4. How will power differentials be addressed in agreements and contracts necessary for the study?
5. How will communities and stakeholders be included in interpreting the findings?

ii. **Advocate for Equitable Access to Funding:** Researchers and funders should use their positionality to establish national and regional funding to diversify access to critical research dollars and backing. This should include advocacy for the ongoing development of funding streams awarded directly to community organizations and making space (e.g., intentionally not applying for certain grants) to redirect power and resources to intervention development teams and organizations led by BIPOC with intersecting identities. Additionally, privileged institutions should share knowledge on successful grant proposals (e.g., best practices, offer to review and edit grants before submission) to more intentionally redistribute power to those who bear the brunt of ineffective HIV prevention and care strategies.
iii. **Build Community Capacity:** Researchers and funders should invest in the development of leadership programs specifically aimed at building the research capacity of communities that are the most marginalized [30]. Organizations like the People Living with HIV and AIDS Leadership Training Institute, the Community HIV/AIDS Mobilization Project Academy, and the AIDS Treatment Activists Coalition are examples of US-based initiatives that support grassroots advocacy and can be used as a catalyst for improving leadership within community [30]. Emphasis should be placed on supporting front-line organizations that have been traditionally underfunded and those that specifically support marginalized communities (e.g., transgender women, people who use drugs, racial and ethnic minorities, men who have sex with men) to provide a more equitable landscape for knowledge creation and encourage the redistribution of power. This also means exploring community-led initiatives that do not necessarily operate under traditional CBO or Non-Governmental Organization constructs.
iv. **Weigh Community Narratives:** Intentional inclusion of qualitative evidence and community narratives should be prioritized alongside quantitative outcomes when weighing the strength and impact of a given intervention. Qualitative data and other narratives provide rich sources of information that contextualize quantitative data in a way that produces more accurate and relevant information in assessing whether the needs of the community are truly being met by a specific EB/EI approach. Prioritizing community voices and perspectives will also create a more intentional collaboration with community rather than merely leveraging their lived experience as “consultants” in the research process. This may mean having to critically reevaluate and potentially deprioritize other aspects of the evidence scoring process that have been traditionally used to measure the strength and validity of an EB/EI approach.
v. **Ensure Equitable Compensation:** Ensure equitable compensation of community members that partner with research institutions and other organizations to develop and implement EB/EI approaches. This means ensuring that each member of a team, not just those directly affiliated with academic institutions, is compensated for their unique contributions to the research process [31]. It also means moving beyond simply establishing an equitable rate of pay and ensuring that community members’ expertise, skills, and resources are fully valued through public acknowledgment and professional opportunities [31]. Equitable compensation paves a way to more equitable partnerships by building mutual trust and respect, sharing power, and providing an avenue for leadership and economic access [31].
vi. **Use Appropriate Language:** Use non-stigmatizing language broadly accepted by the communities centered in the research. It is crucial that the issues defined by EB/EI research, as well as the dissemination of findings, do not inadvertently stigmatize or stereotype certain groups, behaviors, or issues by using language that is not actively used or accepted [29]. PLWH have played a central role in creating inclusive and appropriate dialogue over the last several decades. However, gaps still remain in ensuring that language is oriented towards empowering communities rather than just eradicating disease [32]. Emphasis should be placed on using “people first language,” or language that puts people before a diagnosis or other social condition [29].
vii. **Redefine Expertise:** Convening of expert panels should prioritize community involvement and be approached from a perspective of diversity, inclusion, and equity. Future EB/EI projects should ensure that communities are treated as experts in their own lives and should prioritize this expertise to understand how an intervention will impact their well-being and address ongoing systemic barriers that influence their health outcomes. Additionally, researchers and medical practitioners included as a part of expert committees should represent communities impacted by the research. Organizations and funding entities should include anti-racist experts and community leaders as a part of the core development team when planning EB/EI research initiatives to ensure equity is centered at every stage [33].
viii. **Meaningful Community Integration:** Future EB/EI projects should embed community advisory boards and expert panels/committees that already have community representation. This would allow for a more accurate depiction of what those most impacted would consider relevant, acceptable, and feasible. Community feedback should be weighted as heavily as feedback from academic and medical experts alongside the statistical metrics traditionally used by EB/EI approaches. Incorporating culturally relevant impact metrics will measure HIV-related health outcomes in both reducing systemic and social barriers to care and demonstrating a statistically significant improvement in linkage and retention.

## Conclusion

Public health institutions in the United States have declared racism a public health crisis. To respond to this charge, there must be diversity in the stakeholders responsible for developing effective models needed to end the HIV epidemic in communities where inequities continue to persist despite major advancements in biomedical interventions. Research needs to continue trending towards prioritizing community perspectives in unison with biomedical approaches to capture an ever-evolving cultural landscape and contextualize the effectiveness of interventions over time. EB/EI interventions should be responsive to the varied health and social needs of BIPOC and other historically marginalized groups. The public health community should challenge existing evidence standards and address the ways in which they disfavor community expertise as essential components in “acceptable” science. Moreover, health equity frameworks in HIV research and the implementation of innovative programming should be required by federal and state funders [e.g., utilizing community-based participatory research, the Health Impact Assessment, Public Health Critical Race Praxis, etc.]. That said, it will also be crucial to look beyond academic equity frameworks to truly prioritize community knowledge and agency. This means allowing space for a reframing of expertise to ensure those with lived experience are seen as valuable leaders in the research process rather than passive contributors. It will also require approaching each stage of the EB/EI development and implementation process with a critical eye for power, privilege, and the historical dynamics that exist between the different entities engaged. HIV research innovation offers an opportunity to not only highlight the structures that continue to hinder progress and reinforce inequities in care and prevention outcomes but to break the cycles that keep these structures alive.

## Data Availability

All data and associated materials can be made available for review upon reasonable request.

## Abbreviations

AIDS: Acquired Immunodeficiency Syndrome
ASO: AIDS Service Organizations
BIPOC: Black, Indigenous and People of Color
CBO: Community-Based Organizations
CDC: Center for Disease Control and Prevention
CIE: Center for Innovation and Engagement
EB: Evidence-Based
EHE: Ending the HIV Epidemic
EI: Evidence-Informed
HIV: Human Immunodeficiency Virus
HRSA: Health Resources and Services Administration
LGBTQIA: Lesbian, Gay, Bisexual, Transgender, Queer, Intersex, Asexual
PLWH: People Living with HIV
RFI: Request for Information
SPNS: Special Projects of National Significance
U.S.: United States
